# Application of tumor-node-metastasis staging 2002 version in locally advanced hepatocellular carcinoma: is it predictive of surgical outcome?

**DOI:** 10.1186/1471-2407-10-535

**Published:** 2010-10-07

**Authors:** Binkui Li, Yunfei Yuan, Guihua Chen, Liru He, Yaqi Zhang, Jinqing Li, Guohui Li, Wan Yee Lau

**Affiliations:** 1 State Key Laboratory of Oncology in South China, Guangzhou, China; 2 Department of Hepatobiliary Oncology, Sun Yat-sen University Cancer Center, Guangzhou, China; 3 Department of Surgery, The Third Affiliated Hospital, Sun Yat-sen University, Guangzhou, China; 4 Department of Radiation Oncology, Sun Yat-sen University Cancer Center, Guangzhou, China; 5 Faculty of Medicine, The Chinese University of Hong Kong, Prince of Wales Hospital, Shatin, New Territories, Hong Kong SAR, China

## Abstract

**Background:**

Locally advanced (pT3-4N0M0) hepatocellular carcinoma (HCC) is a heterogeneous group of tumors, which consists of four different categories, including HCC with "multiple tumors more than 5 cm", "major vascular invasion", "invasion of adjacent organs", and "perforation of visceral peritoneum". The aim of our study was to verify whether the 2002 version of the Tumor-Node-Metastasis staging system could predict surgical outcomes in patients with locally advanced HCC.

**Methods:**

We retrospectively reviewed 298 patients with pT3-4N0M0 HCC who underwent hepatic resection from 1993 to 2000 in an academic tertiary hospital. Overall survival (OS) and cumulative recurrence rate (CRR) of the four categories of locally advanced HCC patients were compared.

**Results:**

In multivariate analysis, major vascular invasion was identified as the most significant factor (HR = 3.291, 95% CI 2.362-4.584, *P *< 0.001) followed by cirrhosis status on OS, and was found to be the only independent factor of CRR (HR = 2.242, 95% CI 1.811-3.358, *P *< 0.001) in patients with locally advanced HCC. Among the four categories of locally advanced HCC, OS was significantly worse, and CRR was significantly higher in patients with HCC with major vascular invasion (pT3) than with multiple tumors more than 5 cm (pT3); or tumor invasion of adjacent organs (pT4); or perforation of visceral peritoneum (pT4). No significant differences were observed in OS or CRR between the latter three groups of patients.

**Conclusions:**

HCC with major vascular invasion, which are classified as pT3 under the current TNM staging, have the worst prognosis when compared with the other categories of pT3-4 disease. There is a need to redefine the T classification and to stratify locally advanced HCC.

## Background

Hepatocellular carcinoma (HCC) is one of the most common malignant tumors in the world with a globally increasing annual incidence[1,2]. For accurate prognostic assessment after partial hepatectomy and patient selection for adjuvant therapy, the pathologic tumor-node-metastasis (pTNM) staging system has traditionally been used. This TNM staging system has the advantages of a more detailed T classification than in any other staging systems[3]. However, the current 2002 TNM staging system is not completely satisfactory. The stratification and the prognostic classification of advanced T stages of HCC are most debatable[4-7].

In the 2002 version of the TNM staging system, locally advanced HCC consists of four categories of diseases, with pT3 being classified as multiple tumors more than 5 cm; or tumors with tumor thrombus within the major branch of portal or hepatic veins, and pT4 being classified as tumors with direct invasion of adjacent organs other than gallbladder; or perforation of visceral peritoneum[8,9]. Although major vascular invasion has been recognized as a very strong predictive factor of dismal prognosis,[6,7] and several studies have also shown invasion of adjacent organs or perforation of visceral peritoneum was not definitely associated with a worse survival,[10,11] HCC with invasion of adjacent organs or perforation of visceral peritoneum but not major vascular invasion are classified as pT4 in the current 2002 TNM staging system. These published data strongly suggest HCC in advanced T stages are not classified appropriately under the current TNM classification.

In this study, we retrospectively analyzed the prospectively collected data of 298 patients with pT3-4N0M0 HCC who underwent partial hepatectomy to analyze the ability of the current TNM staging system to predict survival. Overall survival (OS) and cumulative recurrence rate (CRR) of the four categories of locally advanced HCC patients were also compared.

## Methods

### Patients

Between January 1993 and December 2000, 890 patients underwent hepatic resection for HCC with curative intent, which was defined as macroscopically complete tumor resection at Sun Yat-sen University Cancer Center. Three hundred and five (34.3%) patients were classified as locally advanced stages (pT3-4N0M0) according to the 2002 version of American Joint Committee on Cancer (AJCC)/International Union Against Cancer (UICC) TNM staging system[8,9]. Seven patients who died within 30 days of operation were excluded, leaving 298 patients for the analyses.

Preoperative liver functional reserve was assessed by blood biochemistry, Child-Pugh grading, and indocyanine green retention rate at 15 minutes (ICGR15). Only Child-Pugh A patients were offered major hepatic resection, which was defined as the resection of three or more Couinaud's liver segments. In selected Child-Pugh B patients, minor hepatectomy, defined as resection of two or fewer liver segments, was carried out. MELD score was also calculated using pre-operative values of three laboratory tests: INR for prothrombin time, serum total bilirubin and serum creatinine[12]. In our daily practice, the "surgical margin" examination procedure is as follows: the marginal liver tissues taken from the "tumor bed" in the residual liver were used for pathologic review to examine whether it is tumor-free. Both parenchymal involvement of the margin and vascular permeation at the margin were considered as "microscopic positive margin" (which means R1 resection). In the present study, all the tumors had been macroscopically completely resected with a microscopically tumor-free margin proven by the pathologists (which means R0 resection). We define those with an obvious margin as "margin > 0 mm", and the others whose tumor was resected along with the edge of the tumor without an obvious margin but microscopically also with a tumor-free margin as "margin = 0 mm". Tumors with involvement of the ipsilateral branch of the portal or hepatic veins or invasion of adjacent organs were considered resectable provided that en-bloc resection of the entire tumor could be performed with a tumor-free margin. Multiple tumors in more than one hemilivers were resected using extended right or left hepatectomy in patients with adequate hepatic functional reserve (Child-Pugh A and ICGR15 ≤ 10%); otherwise, separate minor hepatectomy of the tumors in the 2 hemilivers were performed.

Histological diagnosis of HCC was reconfirmed by review of pathologic slides. Tumor grade was assessed using the nuclear grading scheme as outlined by Edmondson and Steiner. Tumor size was based on the largest diameter of the tumor in the resected specimen. The number of HCCs was defined by the total number of nodules, including intrahepatic metastasis. Major vascular invasion was defined as gross invasion of the trunk or the main branches of the portal or hepatic veins. Invasion of adjacent organs was defined as gross invasion of an adjacent organ other than the gallbladder which was resected en-bloc with the liver tumor. Perforation into visceral peritoneum was based on gross and histological study of the visceral peritoneum.

This study is a retrospective study of a prospectively collected database. All clinicopathological data were collected at the patient's presentation and at follow-up visits until the patient died or lost to follow-up. We regularly updated the database especially for tumor recurrence and survival status. This study was approved by the Clinical Research Ethics Committee of Sun Yat-sen University Cancer Center and conformed to the ethical guidelines of the 1975 Declaration of Helsinki and the current ethical guidelines. Written informed consent for examination and treatment was obtained from each patient.

### Follow-up

This study was censored on June 30, 2009. The median follow-up was 37 months, (range from 2 to 146 months). At each follow-up visit, we carried out a complete clinical examination of the patient. Serum alpha-fetoprotein (AFP), abdomen ultrasonography and chest x-ray were carried out once every 1-3 monthly in the first year, and once every 3-6 monthly thereafter. When tumor recurrence was suspected, computed tomography and/or magnetic resonance imaging and/or positron emission tomography were done. Whenever possible, salvage treatments were given to patients with recurrence or metastases. The treatments included re-resection, transarterial chemoembolization, radiofrequency ablation, percutaneous ethanol injection and systemic chemotherapy.

### Statistical Analysis

Continuous data were expressed as either mean ± S.D. or medians (range), where appropriate. Chi-square or Fisher's exact test was used to compare the difference of categorical variables. The primary endpoints were overall survival (OS) and cumulative recurrence rates (CRR). OS was defined as the interval from curative surgery to the date of death or the date of last contact if the patient was still alive. Time to tumor recurrence was defined as the interval from surgery to the date when tumor recurrence or metastasis was diagnosed. The survival curves were calculated by the Kaplan-Meier method and compared using the log-rank test. Significant prognostic factors on univariate analysis were entered into a multivariate analysis using the Cox proportional hazards model. A p-value < 0.05 was considered as significant. Statistical procedures were performed using the SPSS software package (Version 15.0; SPSS Inc., Chicago, IL).

## Results

### Demographic and Clinicopathological Data

The demographic and clinicopathological characteristics of the 298 patients with locally advanced HCC in this study are listed in Table 1. In 16 patients, TACE was given followed by hepatic resection after shrinkage of the tumor, and in 76 patients postoperative adjuvant TACE was given. At the time this study was censored, tumor recurrence was diagnosed in 271 patients (90.9%), and 253 patients (84.9%) had died. For the 271 patients with recurrence, 249 patients had intrahepatic recurrence, 15 had both intrahepatic and extrahepatic recurrences, and 7 had extrahepatic recurrence. The 3-year/5-year OS and CRR of the total cohort after hepatic resection were 28.2%/16.9% and 85.7%/90.8%, respectively.

**Table 1:** Clinicopathological characteristics of the whole cohort and four subgroups of patients with pT3/4N0M0 HCC *

Characteristics	Whole cohort (n = 298)	Major vascular invasion (n = 21)	Multiple tumors more than 5 cm (n = 108)	Invasion of adjacent organs (n = 32)	Perforation of visceral peritoneum (n = 57)
Gender, Male:Female	276:22	20:1	100:8	30:2	51:6
Age (years)	48.0 ± 10.7	48.4 ± 12.5	46.1 ± 10.6	49.0 ± 8.2	45.6 ± 10.1
HBsAg positive	257 (86.2%)	17(81.0%)	85(78.7%)	26(81.3%)	51(89.5%)
HCVAb positive	18 (6.0%)	1(4.8%)	3(2.8%)	2(6.3%)	3(5.3%)
ALB (g/L)	42.7 ± 5.4	40.0 ± 4.8	43.1 ± 5.1	39.7 ± 5.7	43.4 ± 5.2
TBIL (μmol/L)	17.0 ± 9.5	18.0 ± 10.9	16.4 ± 9.6	17.6 ± 10.1	16.6 ± 5.9
PT (s)	13.6 ± 1.8	13.9 ± 2.5	13.6 ± 1.9	14.2 ± 2.1	13.0 ± 1.9
ICGR15 (%)	11.1 ± 6.4	12.9 ± 6.8	10.0 ± 5.6	10.3 ± 4.3	8.2 ± 2.8
AFP (μg/L) median (range) ^†^	488 (1-332318)	2839(76-46135)	487(1-332318)	151.5(2-32318)	817(2-266360)
Child-Pugh A	260 (87.6%)	19(90.5%)	97(89.8%)	27(84.4%)	48(84.2%)
MELD score median (range)	7 (6-14)	7(6-9)	7(6-14)	7(6-11)	7(6-10)
Cirrhosis	241 (80.9%)	17(80.9%)	89(82.4%)	26(81.3%)	45(78.9%)
Tumor size (cm)	9.7 ± 3.8	8.6 ± 2.70	9.1 ± 3.0	10.9 ± 3.5	8.8 ± 2.8
Tumor encapsulation	87 (29.2%)	5(23.8%)	36(33.3%)	8(25.0%)	14(24.6%)
Microvascular invasion^‡^	187 (62.8%)	21(100%)	51(47.2%)	20(62.5%)	32(56.1%)
Resection margin > 0 mm	110 (36.9%)	6(28.6%)	39(36.1%)	12(37.5%)	19(33.3%)
Major hepatic resection	217 (72.8%)	16(76.2%)	81(75%)	21(65.6%)	38(66.7%)
Blood loss, liters	0.8 ± 0.5	0.9 ± 0.4	0.8 ± 0.6	0.7 ± 0.4	0.8 ± 0.7
Blood transfusion	157 (52.7%)	14(66.7%)	57(53.3%)	22(68.7%)	36(63.2%)
Previous TACE	16 (5.4%)	1(3.1%)	5(4.6%)	2(6.3%)	2(3.5%)
Postoperative adjuvant TACE	76 (25.5%)	6(28.6%)	32(33.3%)	7(21.9%)	9(15.8%)

Unless otherwise stated, continuous data are expressed as mean ± SD; other figures indicate number of patients.

HBsAg, hepatitis B surface antigen; HCVAb, hepatitis C virus antibody; ALB, albumin; TBIL, total bilirubin; PT, prothrombin time; ICGR15, indocyanine green retention rate at 15 minutes; AFP, α-fetoprotein; MELD, model for end-stage liver disease; TACE, transarterial chemoembolization.

* The four subgroups were selected patients in whom the four pathologic features present singularly.

^† ^Serum AFP levels of patients with major vascular invasion were significantly higher than those of the other three subgroups (*P *< 0.05).

^‡ ^The proportion of patients with microvascular invasion was significantly higher in patients with major vascular invasion than in the other three subgroups (*P *< 0.05).

There were no significant differences between any groups in any other parameters.

### Prognostic Factors for Survival and Recurrence

Among the 24 clinicopathological factors analyzed by univariate log-rank analysis, only albumin (ALB) level (*P *= 0.004), cirrhosis status (*P *= 0.028) and major vascular invasion (*P *< 0.001) were identified as poor prognostic factors for OS (Table 2). The significant prognostic factors found on univariate analysis were further tested in the multivariate Cox model. Major vascular invasion was found to be the most significant factor (HR = 3.291, 95% CI 2.362-4.584, *P *< 0.001), followed by cirrhosis status (HR = 1.400, 95% CI 1.010-1.941, *P *= 0.044). (Table 3)

**Table 2:** Univariate analysis of prognostic factors by log-rank test

Variable	Cases	1y-OS(%)	3y-OS(%)	5y-OS(%)	*P*
Gender					0.913
Female	22	45.5	31.8	18.2	
Male	276	59.1	25.9	15.9	
Age (years)					0.079
≤48	146	54.7	22.9	13.7	
>48	152	60.5	29.6	18.3	
HBsAg					0.269
Negative	41	58.7	37.6	18.8	
Positive	257	56.5	23.2	14.9	
HCVAb					0.380
Negative	280	55.2	27.3	18.2	
Positive	18	47.5	22.0	12.9	
ALB (g/L)					0.004
≤35	37	42.3	7.7	0.0	
>35	261	59.6	27.7	17.3	
TBIL (μmol/L)					0.879
≤20	204	62.8	29.3	16.0	
>20	94	58.0	28.0	14.3	
PT (s)					0.185
≤13.5	148	62.0	32.9	22.3	
>13.5	150	57.0	24.6	14.4	
ICGR15 (%)					0.423
≤10	155	52.2	26.7	17.8	
>10	143	61.7	28.2	19.4	
AFP (μg/L)					0.112
≤25	48	63.2	34.3	20.5	
>25	250	56.0	25.8	16.0	
Child-Pugh					0.275
A	260	59.4	27.4	16.5	
B	38	50.0	18.8	12.5	
MELD					0.243
≤7	169	58.0	24.7	13.9	
>7	129	57.2	32.6	20.7	
Cirrhosis					0.028
No	57	61.0	39.5	26.6	
Yes	241	57.8	23.5	13.8	
Tumor size(cm)					0.278
≤5	35	66.0	31.0	21.0	
>5	263	56.0	23.5	14.5	
**Variable**	**Cases**	**1y-OS(%)**	**3y-OS(%)**	**5y-OS(%)**	***P***
Tumor number					0.101
Solitary	108	59.7	33.3	23.3	
Multiple	190	57.8	23.1	12.5	
Tumor location					0.126
Unilobar	255	52.7	27.2	17.3	
Bilobar	43	45.2	21.4	10.0	
Tumor encapsulation					0.093
Yes	87	54.1	26.1	18.1	
No	211	44.1	17.0	12.0	
Microvascular invasion					0.117
No	111	56.5	24.0	16.7	
Yes	187	48.1	19.4	11.0	
Major vascular invasion					<0.001
No	237	65.2	32.2	19.6	
Yes	61	29.7	0.0	0.0	
Invasion of adjacent organs					0.197
No	219	59.9	27.7	16.0	
Yes	69	56.3	17.0	12.4	
Perforation of visceral peritoneum					0.295
No	220	60.1	26.9	18.4	
Yes	78	53.8	21.0	13.6	
Resection margin(mm)					0.201
0	188	49.3	20.8	12.7	
>0	110	58.4	26.1	17.9	
Resection extent					0.062
Major	217	54.7	23.5	15.2	
Minor	81	67.9	28.0	18.9	
Blood loss(L)					0.140
≤0.8	163	62.8	30.5	21.5	
>0.8	135	52.9	21.2	12.9	
Blood transfusion					0.488
No	142	59.0	26.3	17.6	
Yes	157	57.9	26.6	14.4	
Previous TACE					0.724
No	282	57.3	27.1	16.4	
Yes	16	48.4	21.3	13.2	
Postoperative adjuvant TACE					0.923
No	222	61.7	28.6	15.5	
Yes	76	53.2	23.0	15.2	

**Table 3:** Univariate and multivariate Cox regression analyses for overall survival and cumulative recurrence rate

	Univariate analysis				Multivariate analysis			
	
	OS		CRR		OS		CRR	
	
Variable	HR	*P*	HR	*P*	HR	*P*	HR	*P*
Major vascular invasion*	3.500	<0.001	2.436	<0.001	3.291	<0.001	2.242	<0.001
ALB level ^†^	0.601	0.004	0.541	0.002	0.693	0.094	0.689	0.066
Cirrhosis status ^‡^	1.319	0.028	1.223	0.190	1.400	0.044	-	-

OS, overall survival; CRR, cumulative recurrence rate; HR, hazard ratio.

* Major vascular invasion *vs. *No major vascular invasion

^† ^ALB > 35 g/L *vs. *<35 g/L

^‡ ^Cirrhosis *vs. *No cirrhosis

When the same factors in Tables 2 were analyzed for their prognostic influence on cumulative recurrence rate, only ALB level (*P *= 0.002) and major vascular invasion (*P *< 0.001) were significant factors in the univariate analysis. The 1-year/3-year CRRs of 61 patients with major vascular invasion were 85.2%/100.0%, whereas the corresponding CRRs of 237 patients without major vascular invasion were 54.7%/80.2%, respectively. In multivariate analysis, major vascular invasion was the only significant predictive factor of CRR (HR = 2.242, 95% CI = 1.811-3.358, *P *< 0.001). (Table 3)

### The Impact of Major Vascular Invasion in Stage pT3/4N0M0 HCC

To further verify the grave impact of major vascular invasion on overall survival and recurrence risk in stage pT3/4N0M0 HCC at presentation, we selected patients in whom the four pathologic features were present singularly, and classified them into four subgroups: "Major vascular invasion (n = 21)" as Gp1, "Multiple tumors more than 5 cm (n = 108)" as Gp2, "Invasion of adjacent organs (n = 32)" as Gp3, and "Perforation of visceral peritoneum (n = 57)" as Gp4. Comparisons of clinicopathological data of the four subgroups of patients with pT3/4N0M0 HCC are depicted in Table 1. The four groups were not significantly different in liver function parameters and tumor histological features, except that there was a significantly higher proportion of microvascular invasion in tumors with major vascular invasion than the other three groups. Serum AFP levels were also significantly higher in patients with major vascular invasion compared with the other three groups. Intraoperative blood loss, the proportion of patients requiring blood transfusion, and the proportion of patients receiving preoperative and postoperative TACE were all similar.

**Figure 1:**
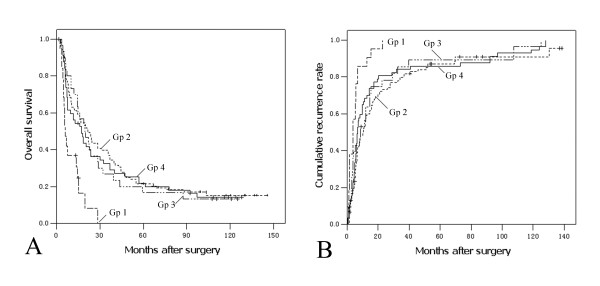
**Kaplan-Meier curves of overall survival and cumulative recurrence rates of four categories of locally advanced HCC patients **. Gp1, major vascular invasion (n = 21); Gp2, multiple tumors more than 5 cm (n = 108); Gp3, invasion of adjacent organs (n = 32); Gp4, perforation of visceral peritoneum (n = 57). (A) Overall survival; Gp1 vs. Gp2, *P *< 0.001; Gp1 vs.Gp3, *P *= 0.001; Gp1 vs. Gp4, *P *< 0.001; Gp2 vs. Gp3, *P *= 0.892; Gp2 vs. Gp4, *P *= 0.693; Gp3 vs. Gp4, *P *= 0.615. (B) Cumulative recurrence rate; Gp1 vs. Gp2, *P *= 0.006; Gp1 vs.Gp3, *P *= 0.001; Gp1 vs. Gp4, *P *< 0.001; Gp2 vs. Gp3, *P *= 0.991; Gp2 vs. Gp4, *P *= 0.299; Gp3 vs. Gp4, *P *= 0.169.

As shown in Figure 1A, the OS of Gp1 (median 5.8 months) was significantly worse than Gp2 (median 21.7 months, *P *< 0.001), Gp3 (median 17.3 months, *P *< 0.001) and Gp4 (median 19.5 months, *P *= 0.001). Similarly, the 3-year CRR of Gp1 (100%) was significantly higher than Gp2 (79.9%, *P *< 0.001), Gp3 (85.5%, *P *= 0.006) and Gp4 (84.2%, *P *= 0.001) (Figure 1B). There were no significant differences in OS or in CRR between the latter three subgroups (*P *> 0.05).

**Figure 2:**
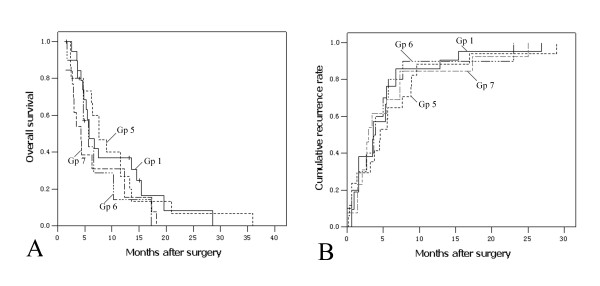
**Kaplan-Meier curves of overall survival and cumulative recurrence rates to compare vascular invasion with or without any of the other pathologic features of locally advanced HCC patients **. Gp1, major vascular invasion (n = 21); Gp5, multiple tumors more than 5 cm and major vascular invasion (n = 17); Gp6, invasion of adjacent organs and major vascular invasion (n = 10); Gp7, perforation of visceral peritoneum and major vascular invasion (n = 13). (A) Overall survival; Gp1 vs. Gp5, *P *= 0.392; Gp1 vs.Gp6, *P *= 0.121; Gp1 vs. Gp7, *P *= 0.901; Gp5 vs. Gp6, *P *= 0.854; Gp5 vs. Gp7, *P *= 0.287; Gp6 vs. Gp7, *P *= 0.250. (B) Cumulative recurrence rate; Gp1 vs. Gp5, *P *= 0.914; Gp1 vs.Gp6, *P *= 0.909; Gp1 vs. Gp7, *P *= 0.455; Gp5 vs. Gp6, *P *= 0.807; Gp5 vs. Gp7, *P *= 0.511; Gp6 vs. Gp7, *P *= 0.651.

To evaluate whether concomitant major vascular invasion influenced survival of patients with other pathologic features, we identified the following three subgroups: "patients with multiple tumors more than 5 cm and major vascular invasion" as Gp5 (n = 17), "patients with invasion of adjacent organs and major vascular invasion" as Gp6 (n = 10), and "patients with perforation of visceral peritoneum and major vascular invasion" as Gp7 (n = 13). Median survivals of Gp5, Gp6 and Gp7 were 7.6 months, 5.7 months and 4.3 months, respectively, while the 3-year CRR of which was all 100%. The OS and CRR of Gp5, Gp6 and Gp7 were compared with Gp1 (major vascular invasion, n = 21). Results showed that there were no significant differences of OS (Figure 2A) or CRR (Figure 2B) among these four subgroups (*P *> 0.05).

### Recurrence Type in Four Groups of Locally Advanced HCC

Since recurrence is the main cause of death after curative partial hepatectomy for HCC, we evaluated the difference in recurrence type for the four categories of locally advanced HCC (Gp1-4). We classified tumor recurrence into early or late with 12 months as the cut-off point,[13,14] and the number of recurrent nodules into single or multiple recurrence. As shown in Table 4, early recurrence occurred in 90.5% of Gp1, which was significant higher than Gp2, Gp3 and Gp4 (*P *= 0.006, 0.041 and 0.024, respectively). Multiple recurrence was observed in 85.7% of Gp1, which was also significantly higher than Gp3 and Gp4 (*P *= 0.039 and 0.036, respectively).

**Table 4:** Recurrence types in four groups of locally advanced HCC

			Recurrence time*			No. of recurrence		
			
Group	Variables	Cases	Early(%)	Late(%)	*P*	Single(%)	Multiple(%)	*P*
Gp1	Major vascular invasion only	21	19 (90.5)	2 (9.5)		3 (14.3)	18 (85.7)	
Gp2	Multiple tumors more than 5 cm only	108	54 (58.7)	38 (41.3)	0.006	32 (34.8)	60 (65.2)	0.067
Gp3	Invasion of adjacent organs only	32	19 (65.5)	10 (34.5)	0.041	12 (41.4)	17 (58.6)	0.039
Gp4	Perforation of visceral peritoneum only	57	34 (64.2)	19 (35.8)	0.024	21 (39.6)	32 (60.4)	0.036

* Early recurrence, recurrence ≤ 12 months; late recurrence, recurrence > 12 months.

## Discussion

To provide a better prognostic prediction for cancer patients, the AJCC/UICC TNM staging system has been updated periodically based on new evidence from clinical studies[15]. In 2002, the TNM staging system for HCC was revised and it put more emphasis on vascular invasion. However, patients with major vascular invasion are still classified as pT3 after this revision. Many studies have shown HCC with major vascular invasion had extremely poor outcomes,[6,7] but HCC with invasion of adjacent organs or perforation of visceral peritoneum were not definitely associated with such a poor prognosis[10,11]. Thus, there is a need to reassess the accuracy of the current TNM classification for pT3-4 tumors.

The issue whether HCC with major vascular invasion is correctly classified was first raised by Poon et al. In their series, patients with major vascular invasion had significantly worse long-term survival compared with the other three categories of stage IVA HCC in the previous version of the TNM classification[7]. In a multicenter study which was the basis of the current TNM staging system, Vauthey et al also showed that the survival of patients with multiple bilobar tumors or invasion of adjacent organs was better than patients with tumors with major vascular invasion[6]. However, a major limitation of their study is that the number of patients with invasion of adjacent organs was relatively small (n = 11, or 2% of the total cohort). Thus, AJCC/UICC decided not to change the definition of pT4 for the 2002 version of the TNM classification. In our present study, major vascular invasion showed the strongest impact on prognosis both in univariate and multivariate analyses. Furthermore, applying the criteria used in the 2002 TNM classification, patients with HCC with major vascular invasion (pT3) in our study had worse survival and more recurrence than HCC with invasion of adjacent organs or perforation of visceral peritoneum (pT4) or multiple tumors more than 5 cm (pT3). Moreover, for patients who had major vascular invasion combined with any of the other three locally advanced pathologic features, the prognosis was similar to patients with HCC with major vascular invasion only. Our results indicated that once major vascular invasion occurred, prognosis became so poor that it was regardless of the presence or absence of any other tumor characteristics. Our data showed there is a need to redefine the T classification and to put more emphasize on major vascular invasion because of its overwhelmingly adverse prognostic influence.

It has also been widely accepted that major vascular invasion indicates advanced HCC with a high risk of recurrence, which is the main cause of death for HCC patients after surgical resection[14,16,17]. In the present study, tumor recurrence was observed in all patients with HCC with major vascular invasion. The incidences of early and/or multiple recurrence for HCC with major vascular invasion was the highest in the four categories of locally advanced HCC, while there were no significant differences in the recurrence types among the remaining three groups of patients. Similar results have also been reported by Portolani et al[16]. In their series, major vascular invasion correlated well with early and diffuse recurrence which was rarely treatable, and thus an unsatisfactory long-term survival. As multiple tumors can be due to multicentric occurrence, they are likely to be associated with a better prognosis[18,19]. The study by Poon et al. showed early recurrence within one year after resection of HCC was more likely to be due to metastasis than multicentric occurrence and was associated with a worse prognosis when compared with late recurrence[20]. Our results supported that patients with HCC with major vascular invasion had a significantly poorer prognosis than those with multiple tumors more than 5 cm, those with invasion of adjacent organs, or those with perforation of visceral peritoneum.

The prognosis of HCC is extremely poor in patients with advanced disease[21]. For hepatic resection, the presence of distant metastasis is generally considered a contraindication[22]. In contrast, in view of the lack of other effective treatment options, surgical resection especially in eastern countries, is still advocated as the treatment of choice for patients with locally advanced HCC which is classified as pT3-4N0M0[22-24]. Ishizawa et al. demonstrated that partial hepatectomy provided survival benefits for patients with multiple tumors with Child-Pugh class A cirrhosis[25]. For locally advanced HCC, studies have also shown that survival following hepatic resection compared favorably with those treated non-surgically[10,11,26,27]. To improve future survival rates, innovative multidisciplinary approaches will also be needed. With recent advances in adjuvant therapy such as immunotherapy and molecular targeted therapy, whether these new modalities can improve the prognosis of locally advanced HCC treated with curative resection need to be clarified[28,29].

For patients with major vascular invasion, the prognosis is particularly grave. Nonsurgical treatment with systemic chemotherapy, intra-arterial chemotherapy, or radiofrequency ablation for these patients results in dismal 1-year survival rates, ranging from 7% to 18%[30-32]. Patients with HCC and major vascular invasion do not present technical contraindications to surgery. However, results after hepatic resection for HCC with major vascular invasion have been disappointing[22,33,34]. In the current study, the median OS was only 5.8 months after hepatic resection. Recently, the efficacy of Sorafenib in patients with advanced HCC has been evaluated in a randomized, double-blind, multicentre, phase III trials: the SHARP (Sorafenib Hepatocellular Carcinoma Assessment Randomized Protocol) trial[35]. However, the placebo group of the SHARP study with similar baseline characteristics as in this study had a median survival of 7.9 months. It seems that these patients may not benefit from resection or even suffer detrimental effects. Since there are no head-to-head studies comparing resection and nonsurgical treatment or sorafenib in this category of patients, future studies will need to investigate more thoroughly which modality should be used as first line treatment.

There are several limitations of our study. Our study is retrospective and it is based on a single-institutional experience. The sample size of some of the subgroups is relatively small. A larger scale, multi-center study is needed to confirm our results.

## Conclusion

In conclusion, our study suggested that the current 2002 TNM staging system failed to stratify patients with different categories of locally advanced HCC according to the outcomes after hepatic resection. The current pT3 subgroup of major vascular invasion exhibited poor surgical outcomes when compared with the other pT3 subgroups with multiple tumors more than 5 cm, and the pT4 group with invasion of adjacent organs or perforation of visceral peritoneum. These data, in addition to the published data, challenge the value of the current 2002 TNM staging classification in locally advanced HCC.

## Competing interests

The authors declare that they have no competing interests.

## Authors' contributions

BKL, YFY, JQL, and GHL have made substantial contributions to conception and design of the study. BKL, GHC, and YQZ carried out acquisition of data. BKL, GHC, and LRH carried out analysis and interpretation of data. BKL, YFY, LRH and WYL have been involved in drafting the manuscript. All authors read and approved the final manuscript.

## Pre-publication history

The pre-publication history for this paper can be accessed here:

http://www.biomedcentral.com/1471-2407/10/535/prepub
